# Milky Plasma, Murky Diagnosis: Urgent Plasma Exchange for Severe Hypertriglyceridemia‐Induced Hyperviscosity Without Pancreatitis, but With Myocardial Infarction

**DOI:** 10.1002/jca.70095

**Published:** 2026-02-18

**Authors:** Catherine Bodnar, Christina Hughey, Ariel Bodker, Chinelo P. Onyenekwu

**Affiliations:** ^1^ Department of Pathology and Laboratory Medicine University of Wisconsin‐Madison Madison Wisconsin USA; ^2^ Department of Medicine University of Wisconsin‐Madison Madison Wisconsin USA

**Keywords:** acute coronary syndrome, apheresis indications, cardiorespiratory compromise, hyperviscosity syndrome, microvascular flow, therapeutic apheresis, triglycerides

## Abstract

Severe hypertriglyceridemia can increase the risk of acute pancreatitis, but also clinically significant hyperviscosity syndrome characterized by the typical signs of neurologic and visual manifestations. Hyperviscosity syndrome is a well‐established medical emergency. However, hyperviscosity syndrome induced by hypertriglyceridemia may be under‐recognized, particularly when pancreatitis and other classical signs of hyperviscosity syndrome are absent. We describe an index case of extreme hypertriglyceridemia presenting with cardiorespiratory symptoms (chest pain and dyspnea) due to suspected hyperviscosity, but without clinical or radiographic evidence of pancreatitis, successfully treated with therapeutic plasma exchange (TPE). We then review the limited, but growing body of published case reports describing TPE for hypertriglyceridemic hyperviscosity without acute pancreatitis. Across cases, TPE reliably achieved rapid triglyceride reduction and resolution of hyperviscosity symptoms. This review highlights diagnostic challenges, therapeutic indications, and the rationale for early TPE in symptomatic hyperviscosity even when pancreatitis is not present.

## 1 Introduction

Hypertriglyceridemia is a metabolic disorder fundamentally characterized as a persistent elevation of serum triglyceride levels. The disorder is influenced by a constellation of familial/genetic, lifestyle, and environmental factors, which cause significantly variegated phenotypic expression with clinical consequences that include pancreatitis and atherosclerotic cardiovascular disease [1]. Severe hypertriglyceridemia described as serum triglycerides greater than 1000 mg/dL, is a well‐established cause of acute pancreatitis [2, 3]. Less widely appreciated, however, is the capacity of severe hypertriglyceridemia to induce hyperviscosity syndrome [4, 5], a rheologic disorder caused by markedly lipemic, chylomicron‐rich plasma. Currently, the American Society for Apheresis' 2023 guidelines on the use of therapeutic apheresis considers only hypertriglyceridemic pancreatitis as an indication (Category III) for therapeutic plasma exchange (TPE) [6]. A category III classification specifies that the literature has not yet established the optimum role of apheresis for a particular disorder, such that clinical decisions must be made on an individual basis [6]. The ASFA guidelines include TPE as first line (Category I) treatment for hyperviscosity syndrome. However, this medical emergency is listed only in association with blood malignancies causing hypergammaglobulinemia—most commonly Waldenström macroglobulinemia and multiple myeloma—and not for the elevation of other substances in plasma/serum such as lipids [2]. Due to the Category I classification and the potentially life‐threatening nature of symptomatic hyperviscosity in hypergammaglobulinemia, TPE is usually performed emergently upon recognition of the condition [7, 8, 9].

Classically, hyperviscosity syndrome presents as a triad of visual changes, sudden neurological/central nervous system deficits, and mucosal bleeding [4, 10]. Very rarely, hyperviscosity syndrome can lead to cardiopulmonary manifestations, such as high‐output heart failure or acute coronary syndrome (ACS), which may occur from a non‐hematological etiology [2, 4, 5]. In hyperviscosity syndrome caused by hematological malignancy, apheresis is emergently utilized to physically remove the causative substance from the serum (e.g., immunoglobulins), thereby lowering the blood's internal resistance to flow and improving microcirculation and perfusion of tissues by lowering serum concentration of the immunoglobulins [5]. In theory, apheresis could be used to treat hyperviscosity caused by the elevation of other substances in the serum, such as lipids, by this same mechanism.

We report an unusual case of hypertriglyceridemia‐induced hyperviscosity syndrome presenting with an atypical clinical presentation of ACS, in the absence of acute pancreatitis. We provide a brief review of the available literature on this uncommon but clinically significant entity, highlighting its diagnostic challenges and the efficacy of TPE in its management. This case suggests the efficacy of plasma exchange for severe hypertriglyceridemia without pancreatitis, but with myocardial infarction due to hyperviscosity syndrome.

## 2 Case History

A 55‐year‐old man with a history of renal transplant, familial hyperlipoproteinemia type V, severe hypertriglyceridemia complicated by numerous episodes of pancreatitis, pulmonary emboli on apixaban, heart failure with preserved ejection fraction, and type II diabetes mellitus presented with 1 week of progressive exertional substernal chest pain with radiation to the left upper extremity and associated shortness of breath. A cardiac catheterization completed 1 year prior revealed no evidence of significant obstructive coronary disease and only luminal irregularities. Of note, the patient had also previously undergone 2.5 years of maintenance therapeutic plasmapheresis (approximately two decades prior to his current presentation) for recurrent pancreatitis in the setting of severe hypertriglyceridemia and had subsequently been transitioned to medical therapy for management of his dyslipidemia. The patient had been taking Fenofibrate, Icosapent Ethyl, Rosuvastatin, and Ezetimibe daily prior to presentation, with no adherence issues identified.

On admission, the patient was dyspneic and continued to report chest pain, but he denied abdominal pain or symptoms of pancreatitis. His venous blood specimen at bedside was chylous. His laboratory test results were remarkable for elevated triglyceride level of 5286 mg/dL (reference: < 150 mg/dL), total cholesterol of 679 mg/dL (reference < 200 mg/dL), troponin 0.12 ng/mL (reference ≤ 0.03 ng/mL), and serum lipase 14 U/L (reference: ≤ 60 U/L). Serum viscosity was noted to be elevated at 2.01 cP (normal: ≤ 1.50 cP). Computed Tomography scan of the abdomen was unremarkable with no acute findings of pancreatitis and without evidence of peripancreatic inflammation. Chest radiograph was non‐contributory. Subsequent electrocardiogram (ECG) showed T wave inversions in lead I. Overall, findings were assessed to be consistent with ACS in the setting of severe hyperviscosity syndrome.

Given this clinical presentation and supporting laboratory data, insulin and heparin drips were initiated for presumed unstable angina/possible non‐ST elevation myocardial infarction (NSTEMI) in the setting of hyperviscosity syndrome due to severe hypertriglyceridemia. He continued all his lipid‐lowering medications throughout the hospital admission. However, over 12 h, only a marginal reduction in triglyceride levels (4795 mg/dL) was achieved with this combination of medical therapies. The patient also continued to endorse severe substernal chest pain and dyspnea.

The apheresis service was consulted to consider TPE for severe hypertriglyceridemia. A decision was made to urgently initiate TPE for the rapid reduction of serum triglycerides. Daily therapeutic apheresis procedures were completed over 3 days. Plasma exchange was performed using an automated, continuous‐flow centrifugation platform, the Spectra Optia Apheresis System (Terumo BCT Inc., Lakewood, CO, USA), version 12, with the TPE software application. Vascular access was via a right internal jugular (IJ) vein non‐tunneled, dual‐lumen apheresis catheter. One plasma volume was exchanged at each procedure, using 5% human serum albumin as the primary exchange fluid, while finishing the first two apheresis procedures with 3 units of fresh‐frozen plasma. The whole blood to anticoagulant (Acid Citrate Dextrose‐Adenine—ACD‐A) ratio was set at 8:1, and the inlet flow rate was set at 75 mL/min. The patient was managed on an intermediate‐care floor with continuous cardiac telemetry. Vital signs during the exchange procedure were captured directly from his telemetry monitor data. Mid‐procedure during the first TPE, the original inlet line became nearly completely occluded and could not be cleared despite repeated flushing attempts. Therefore, a switch was made such that the initial IJ catheter return line became the inlet. An 18G right forearm peripheral intravenous line was then used for the return for the rest of the procedure, with success. After the first two TPE procedures, the apheresis catheter was flushed, locked, and capped with 2 mL of Alteplase. The remaining two TPEs were performed successfully with vascular access via the non‐tunneled central venous apheresis catheter. Aside from the initial challenge with vascular access, the patient experienced no TPE‐related side effects or complications during the procedures. Triglyceride levels roughly halved after each consecutive apheresis procedure, with 89% reduction at the end of the third procedure (2091 mg/dL after the first, 1267 mg/dL after the second, and 516 mg/dL after the third, as shown in Figure S1). Waste bags after each apheresis procedure were notable for significant milky plasma with a visual improvement in turbidity after each procedure (Figure S2 shows the waste bag after the first TPE). Intravenous infusions of heparin and insulin were discontinued after the second TPE. The patient tolerated apheresis well with significant alleviation of cardiorespiratory hyperviscosity syndrome‐related symptoms, concurrent downward trends in troponin, and resolution of the dynamic T‐wave changes on ECG.

## 3 Discussion

Treatment of severe hypertriglyceridemia without pancreatitis, but with hyperviscosity syndrome by therapeutic apheresis is a relatively rare clinical scenario documented in literature. A structured literature search was conducted in PubMed/MEDLINE, Embase, and Web of Science from inception to November 25, 2025, using combinations of terms for hypertriglyceridemia, hyperviscosity syndrome, TPE, plasmapheresis, and lipoprotein apheresis. Filters were applied for human case reports. Articles were excluded if acute pancreatitis was the primary TPE indication, no symptoms or description of hyperviscosity were provided, or apheresis was not used. Only three eligible published human case reports were identified (Table 1) [11, 12, 13]. Unique to our case is the unusual presentation of hyperviscosity syndrome with only cardiorespiratory symptoms in the absence of neurologic or visual disturbances or mucosal bleeding. Across the index and published cases, common features included serum triglyceride levels > 5000 to 10 000 mg/dL; symptoms reflecting microvascular sluggish flow, such as headache, photophobia, blurred vision, dizziness, fatigue, and, in the index case, dyspnea and chest pain; and the absence of pancreatitis. Clear triggers include biologic therapy (Risankizumab) [12], mammalian target of rapamycin (mTOR) inhibition (sirolimus) [13], and excessive lipid infusion for local anesthetic systemic toxicity [11]. The index case was thought to be precipitated by dietary indiscretion.

**TABLE 1:** Summary of evidence for therapeutic plasma exchange in hypertriglyceridemia‐associated hyperviscosity without pancreatitis.

Author (year)	Diagnosis	Peak TG (mg/dL)	Acute pancreatitis present	Hyperviscosity features	Number of apheresis procedures and replacement fluid	Outcome
Bodnar (Index Case)	Familial hyperlipoproteinemia type V	5286	No	Unstable angina, Dyspnea	Three; albumin and plasma	TG drop and improvement in symptoms
Yasgur (2025)	LAST → lipid emulsion toxicity	> 10 350	No	Headache, photophobia, blurry vision	One; plasma	Rapid TG drop, symptom relief
Abdulwadood (2024)	Risankizumab‐induced HTG	7670	No (normal CT)	Headaches, chest pain	Two; ^a^	TG drop, resolution of symptoms
Bender (2022)	Sirolimus HTG	12200^b^	No	Neurologic signs Pink/milky blood	Five; albumin	Viscosity improved

Abbreviations: CT, computerized tomography; HTG, hypertriglyceridemia; LAST, local anesthetic systemic toxicity; TG, triglycerides.

^a^ Replacement fluid not reported.

^b^ Converted unit.

Our review of the available literature underscores that severe hypertriglyceridemia can cause clinically significant hyperviscosity syndrome even in the absence of pancreatitis, and that TPE is an effective, rapid therapeutic modality in this context. This index case expands the phenotype by demonstrating cardiorespiratory hyperviscosity symptoms of dyspnea and chest discomfort without pulmonary pathology or obstructive cardiovascular disease. These symptoms resolved following TPE, highlighting the central role of microcirculatory dysfunction in symptom generation. Given the category III status and the similar efficacy of insulin and TPE, some apheresis physicians consider TPE for hypertriglyceridemia‐induced pancreatitis only if triglycerides remain very high after at least 24–48 h of insulin therapy [14].

Hyperviscosity syndrome is a true medical emergency characterized by elevated serum viscosity due to an inordinate elevation in the serum concentration of a particular blood component (red blood cells, white blood cells, platelets, or acellular components such as serum proteins or lipids). Most commonly, hyperviscosity syndrome manifests secondarily to hypergammaglobulinemia in the setting of certain hematologic malignancies, such as Waldenström macroglobulinemia and multiple myeloma [4]. Classic symptomatology of hyperviscosity syndrome includes a triad of neurological deficits/headaches, vision deficits, and mucosal bleeding, though rarer manifestations of priapism and ACS have also been described [4, 15]. Interestingly, the index patient exhibited none of the classic triad of symptoms of hyperviscosity syndrome. Rather, the main presenting complaint was cardiac in nature. While a quick superficial review of the patient's history may lead to the conclusion that the patient's angina was due to presumed atherosclerotic cardiovascular disease alone in the setting of metabolic syndrome, long‐standing hypertriglyceridemia, and type 2 diabetes mellitus, the patient's relatively recent coronary angiogram without obstructive coronary disease, coupled with a severely elevated serum triglyceride level, and an elevated plasma viscosity measurement argue against this conclusion. These findings instead suggest that the angina was secondary to hyperviscosity caused primarily by the patient's high serum lipid levels, a phenomenon previously described in literature [8]. The resultant hyperviscosity from severe hypertriglyceridemia caused a microvascular dysfunction resulting in an oxygen mismatch and reduced oxygen delivery to the tissues [16]. Few clinicians consider hyperviscosity in hypertriglyceridemia unless visual or neurologic symptoms predominate. Our case and published literature [15] illustrate that cardiopulmonary manifestations may be equally important and require prompt lipid clearance.

Indeed, elevated triglycerides have been shown to increase plasma viscosity, with at least one study measuring the contribution of plasma triglycerides to overall blood viscosity after adjusting for contributions by other major covariates like hematocrit and serum protein [5]. In turn, hypertriglyceridemia‐induced elevated blood viscosity causes increased propensity for atherothrombosis over time, through multifactorial means that include shear stress of the endothelium, impairment of microcirculatory blood flow, and plasma protein interactions with post‐stenotic vascular recirculation zones [17].

In the acute setting, severely elevated serum triglyceride levels such as can be found in familial forms of hypercholesterolemia and hypertriglyceridemia can cause a clinical manifestation of true hyperviscosity syndrome, though documentation of this phenomenon in literature has been sparse [16]. Such was the case with this patient, who presented with hyperviscosity syndrome manifesting as ACS in the setting of an acute exacerbation of his type V familial hyperlipoproteinemia. Clinical and laboratory evidence of myocardial injury coupled with the lack of response to medical therapy directed clinical decision‐making to therapeutic apheresis as a treatment modality despite the absence of concomitant pancreatitis. We supplemented replacement fluid with plasma in the initial two sessions while the patient received heparin. Plasma serves as a source of lipoprotein lipase, which is central in the metabolic clearance of triglycerides from chylomicrons and is depleted by heparin.

Viscosity measurements are rarely available emergently and were not included in the reviewed cases described above. This index case is unique in that available laboratory viscosity results provide further insight into the pathophysiology of the disease process. However, clinical diagnosis relies on pattern recognition, visualization of lipemic serum, severe elevation in triglyceride levels, and symptom resolution following TPE.

The dramatic results, both in terms of lowered triglyceride levels and in resolution of the patient's symptoms, suggest an alternate, expanded role for therapeutic apheresis in the setting of hypertriglyceridemia‐induced hyperviscosity syndrome and in cases of hypertriglyceridemia without pancreatitis. Symptomatic hyperviscosity, regardless of etiology, should be considered a stand‐alone indication for early TPE analogous to hyperviscosity caused by gammopathies. Furthermore, hypertriglyceridemia in the absence of pancreatitis may benefit from TPE in addition to hypertriglyceridemic pancreatitis.

## Funding

The authors have nothing to report.

## Disclosure

The authors have nothing to report.

## Ethics Statement

This case report did not require institutional review board approval. Informed consent for publication of de‐identified clinical information was obtained from the patient.

## Conflicts of Interest

The authors declare no conflicts of interest.

## Supporting information


**Figure S1:** Trend in serum triglyceride levels with TPE treatment.
**Figure S2:** Therapeutic plasma exchange waste bag after the first apheresis procedure showing milky colored waste plasma.

## Data Availability

The data supporting the findings of this case report are contained within the article. No additional datasets were generated.
