# Supplementary material for: Milky Plasma, Murky Diagnosis: Urgent Plasma Exchange for Severe Hypertriglyceridemia‐Induced Hyperviscosity Without Pancreatitis, but With Myocardial Infarction
Source: J Clin Apher. 2026 Feb 18;41(1):e70095. doi: 10.1002/jca.70095 (PMC12917290; doi:10.1002/jca.70095)

Supplementary Figure 1. Trend in Serum Triglyceride Levels with TPE Treatment


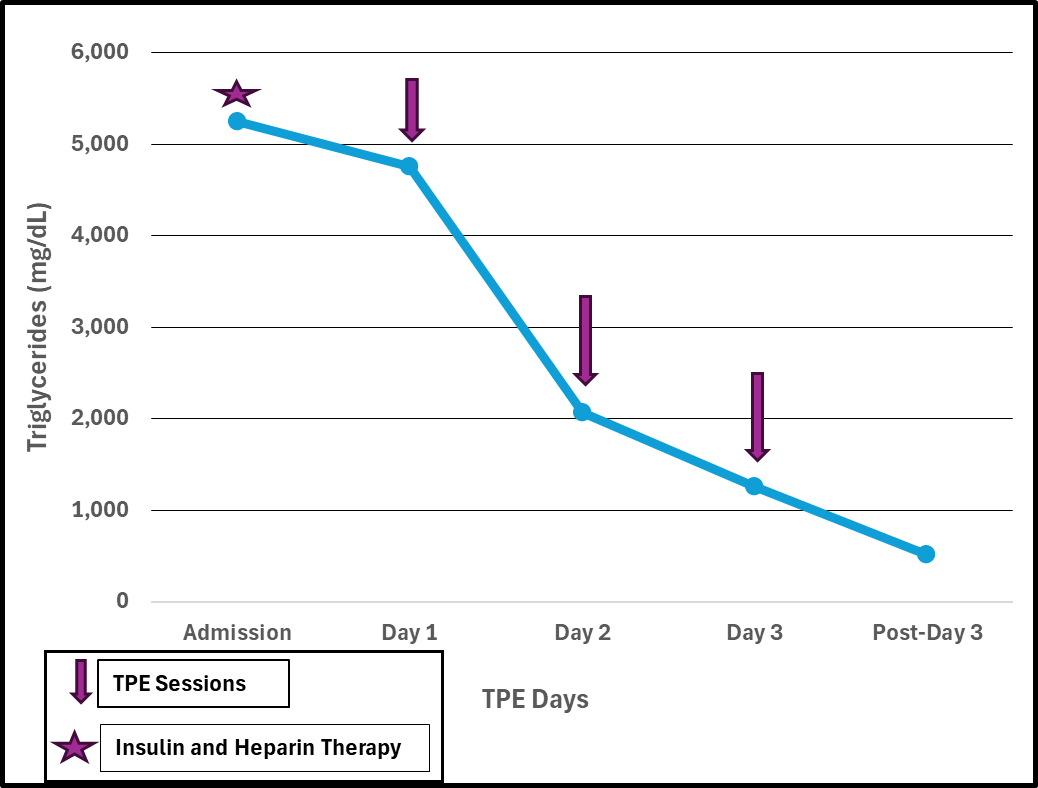


Intravenous Insulin and Heparin were discontinued by day 2. TPE- Therapeutic plasma exchange.

Supplementary Figure 2. Therapeutic Plasma Exchange Waste Bag after the First Apheresis Procedure Showing Milky Colored Waste Plasma.


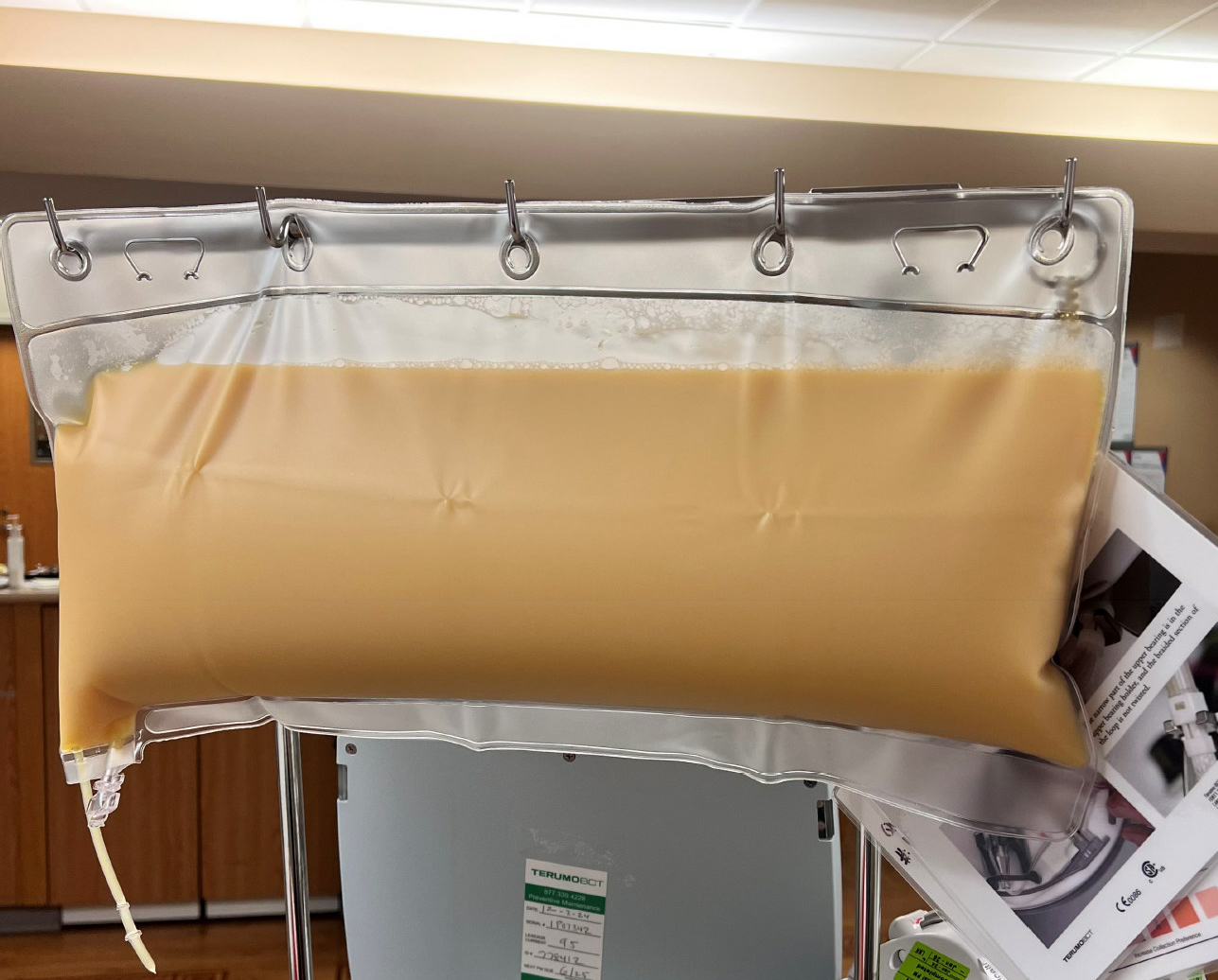

Supplement: Supplementary file 1 — Figure S1: Trend in serum triglyceride levels with TPE treatment. Figure S2: Therapeutic plasma exchange waste bag after the first apheresis procedure showing milky colored waste plasma. [file JCA-41-e70095-s001.docx]
